# A simple and novel way of maintaining constant wall temperature in microdevices

**DOI:** 10.1038/srep18230

**Published:** 2016-01-22

**Authors:** V. S. Duryodhan, Abhimanyu Singh, Shiv Govind Singh, Amit Agrawal

**Affiliations:** 1 Indian Institute of Technology Bombay, Powai, Mumbai 400076, India; 2 Indian Institute of Technology Hyderabad, Hyderabad 502205, India

## Abstract

Constant wall temperature /homogeneity in wall temperature is the need of various lab-on-chip devices employed in biological and chemical investigations. However method to maintain this condition does not seem to be available. In this work, a novel and simple way of maintaining constant wall temperature is proposed. A diverging microchannel along with conjugate effects is utilized towards this end. Both measurements and three dimensional numerical simulations are undertaken to prove the design. The investigation has been carried out over a large parameter range (divergence angle: 1–8°; length: 10–30 mm; depth: 86–200 μm; solid-to-fluid thickness ratio: 1.5–4.0, and solid-to-fluid thermal conductivity ratio: 27–646) and input conditions (mass flow rate: 4.17 × 10^−5^ −9.17 × 10^−5^ kg/s, heat flux: 2.4–9.6 W/cm^2^) which helped in establishing the finding. It is observed that a nearly constant wall temperature condition can be achieved over a large parameter range investigated. A model to arrive at the design parameter values is also proposed. The method is further demonstrated for series of microchannels where we successfully maintain each station at different temperature within ±1 °C. The finding is therefore significant and can be employed in both single and multi-stage processes such as PCR requiring different constant wall temperature with a fine resolution.

The primary objective of this study is to propose a method for maintaining constant wall temperature in microdevices. In several biological applications, it is required to maintain the cells above/ below the room temperature implying the need for heating/ cooling the microdevice. For instance, Hung *et al.*^1^ designed a microfluidic cell culture array for studying the human carcinoma cells in which a constant temperature of 37 °C was required to be maintained. Wan *et al.*^2^ studied proliferation and migration of tumor cells under a controlled temperature of 37 °C. Polymerase chain reaction (PCR) is a widely employed technique to amplify a particular DNA sequence and is used in a number of applications like pathogen detection^3^,^4^ and hereditary disorder diagnosis^5^. The entire process takes place in three steps: denaturation, annealing and extension for which the different stations are required to be maintained at constant temperatures of 95, 55 and 72 °C, respectively. Selva *et al.*^6^,^7^ required a certain temperature gradient in order to control the movement of bubbles and droplets by means of thermocapillary effects. These examples underline the need for developing an effective and efficient system which could maintain the microchannel walls at a desired temperature.

When the temperature of the sample is to be raised, as in the above examples, one can either supply a uniform heat flux at the boundaries, or maintain the boundaries at a higher temperature. As is well known, for hydrodynamically and thermally fully-developed flow in simple geometries, a uniform heat flux leads to a linear increase in surface temperature, and a constant difference between the bulk fluid and wall temperatures. The presence of a lateral temperature gradient in the fluid however implies that the local fluid temperature can exceed the safe operating temperature limit. Maintaining the boundaries at a constant temperature is therefore the safer alternative where the local fluid temperature will necessarily be less than the wall temperature (in the wall heated case). At the conventional scale, the constant wall temperature condition is typically maintained by employing phase change^8^,^9^. Maintaining constant temperature is however not simple, more so when extended to microscale. Moreover, only certain temperatures (and not any desired temperature) can be achieved with the phase change technique due to the limitations in the material that can be employed for this purpose.

Hung *et al.*^1^ and Wan *et al.*^2^ employed an incubator to maintain constant surface temperature. The primary limitation of the approaches in^1^,^2^,^3^,^4^,^5^ is that the device employed is bulky and cannot be readily integrated with a microdevice. Few research groups have tried to control the temperature gradients at microscale by using active control strategies. For example, Khandurina *et al.*^10^ used an integrated system for PCR analysis in which a compact thermal cycling assembly based on dual Peltier thermoelectric elements coupled with a microchip gel electrophoresis platform is employed. Hsieh *et al.*^11^ and Wang *et al.*^12^ employed an array type of microheater with an active compensation unit. This is in contrast with the conventional block heater employed to achieve constant wall temperature for PCR application. Persat and Santiago^13^ employed an unconventional way of chemical cycling (reducing the melting temperature of DNA with addition of solvent) which reduces thermal cycling. Wu *et al.*^14^ fabricated a microheater and a thermal sensor inside a PDMS microchannel using injection molding and demonstrated uniformity in temperature distribution with the help of high precision control system. Shaw *et al.*^15^ employed a microwave based heating in combination with air impingement cooling for performing PCR in a microfluidic device. Kim *et al.*^16^ used microdevice with controlled heater power for reverse transcription RT-PCR process to analyze influenza A H1N1 virus. Their device was integrated with an on-chip colorimetric immuno-chromatographic strip. Miralles *et al.*^17^ summarized the various techniques till date used for heating and cooling of microdevices along with their applications and limitations.

The level of complexity associated in the earlier approaches is noteworthy. This defeats the primary reason of employing a microdevice in the first place. In this paper, we propose a much simpler solution to this problem.

## Proposed Design

**Figure 1:**
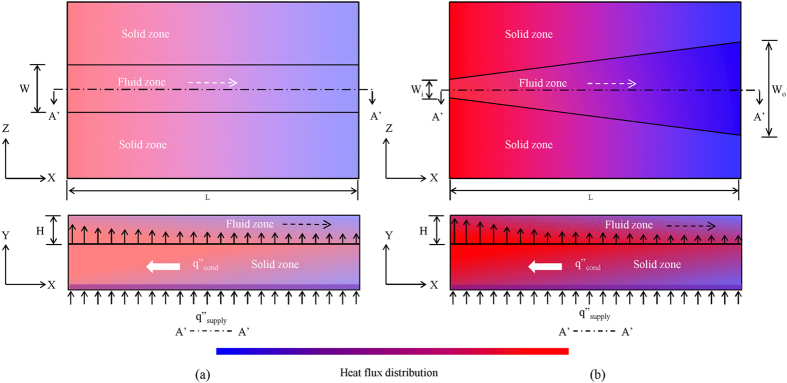
Schematic representation of proposed method to bring constant wall temperature condition (**a**) Uniform cross section microchannel, (**b**) Diverging microchannel.

An effect that becomes particularly relevant at the microscale is conduction in the walls (or conjugate effect), which leads to a redistribution of heat flux and temperature in both the flowing fluid and the solid walls^18^,^19^,^20^,^21^. At the microscales, the characteristic dimensions of the microchannel are of the same order or may be smaller than the thickness of the substrate. When such a situation arises, the axial conduction through the solid cannot be neglected; heat transfer then happens by convection in the fluid coupled with axial conduction in the solid. In several applications, the bottom wall of the substrate is subjected to a constant heat flux boundary condition. If wall conduction comes into the picture then heat flux at the bottom of the microchannel (fluid-solid zone interface) is no longer constant (Fig. 1a). (The color-map legend in Fig. 1 shows the heat flux distribution.) This redistribution in heat flux alters the surface temperature, resulting in a reduced surface temperature gradient compared to that for uniform heat flux boundary condition. It is noteworthy that heat flux can be further redistributed by employing geometry of varying cross section (Fig. 1b). The idea presented in this work makes use of these two effects (wall conduction and varying cross section) in order to reduce the surface temperature gradient in a diverging microchannel.

To prove this concept, heating in diverging microchannel is undertaken with DI water as the working fluid. The bottom of the substrate is subjected to a constant heat flux boundary condition. A comprehensive study of the ensuing temperature distribution at the substrate bottom of the diverging microchannel is carried out experimentally as well as numerically. A detailed parametric study has also been performed at various geometrical, thermo-physical and input conditions in order to understand their effect on the bottom wall boundary condition. A model is also proposed to obtain the value of the surface temperature as a function of various governing parameters. Finally, the design is demonstrated to achieve three different isotherm surfaces in series which is useful for PCR application.

## Results and Discussion

### Proof of the proposed design

The combined results from experimental and numerical investigations will be presented and discussed in this section. First, the surface temperature distribution in an 8^o^ diverging microchannel was compared with a straight microchannel having a uniform width of 1133 μm, depth of 86 μm, same solid thickness and material. Note that the width of uniform cross section microchannel has been selected based on the concept of equivalent hydraulic diameter^22^. The simulation was carried out at a mass flow rate of 8.33 × 10^−5^ kg/s (5 ml/min) with a constant heat flux of 4.8 W/cm^2^ supplied at the bottom wall.

**Figure 2:**
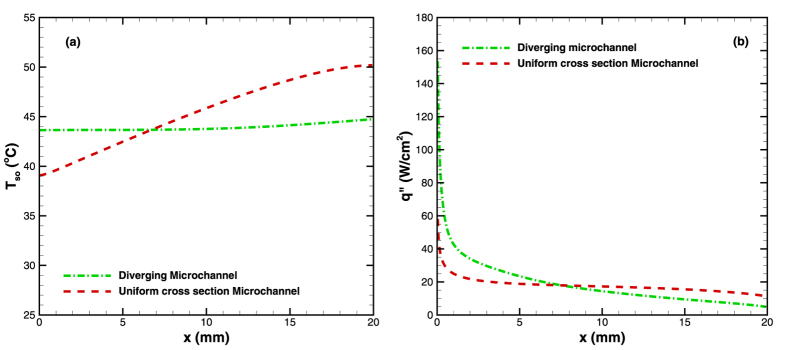
Comparison of (**a**) surface temperature, (**b**) heat flux distribution in a diverging and a straight microchannel obtained numerically.

**Figure 3:**
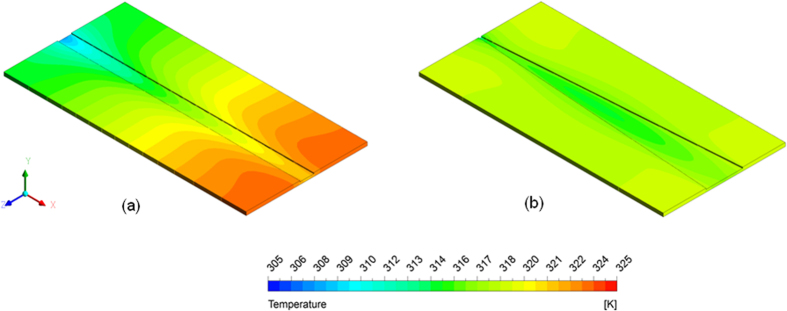
3D contour plots for temperature in (**a**) uniform cross sectional microchannel and (**b**) diverging microchannel obtained numerically.

Figure 2a shows that the surface temperature difference between the inlet and outlet was significantly higher for straight microchannel (11 °C) as compared to a diverging microchannel (1 °C). A constant heat flux (*q”*) is imposed at the outer bottom surface of the microchannel (i.e. heater) suggesting the constant per unit heat input (q/L) to the inner surface. The perimeter *P*(*x*) of diverging microchannel is however a function of flow direction, which leads to a varying heat flux 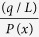 along the flow direction. Therefore, in the case of diverging microchannel, the heat flux magnitude at the inlet is higher since the perimeter at the inlet is smaller (Fig. 2b). This leads to an increase in the surface temperature near the inlet of diverging microchannel (Fig. 2a). The perimeter of the microchannel keeps on increasing as we move downstream which leads to a decrease in the heat flux absorbed. Hence, the surface temperature near the outlet side is reduced to some extent (Fig. 2a). Also, the temperature gradients in the solid created by the flowing liquid lead to an axial back heat conduction which tries to reduce the temperature gradient. We therefore find that the temperature rise in a diverging microchannel is much less than a straight microchannel under identical conditions. This is also shown for entire substrate through 3D contour plots for temperature in Fig. 3. This result is further verified through experiments.

**Figure 4:**
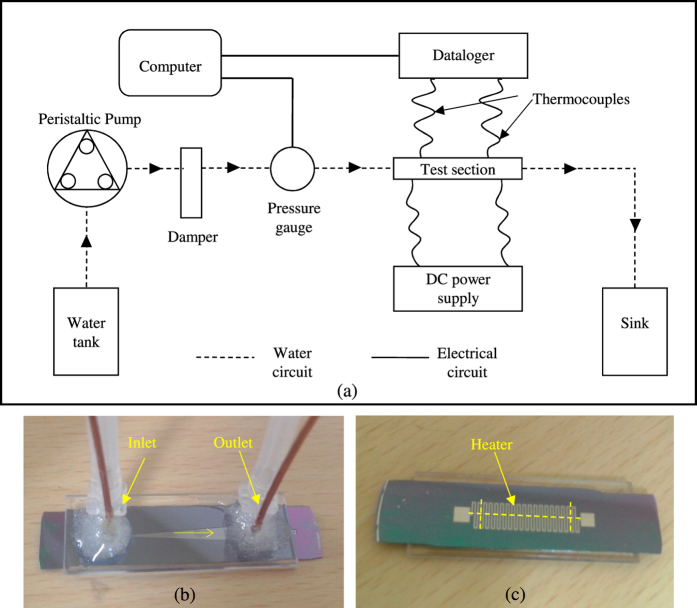
(**a**) Schematic of the experimental setup, (**b**) top and (**c**) bottom view of test section. The dashed line in Fig. 4c shows the path along which local temperatures were measured.

**Figure 5:**
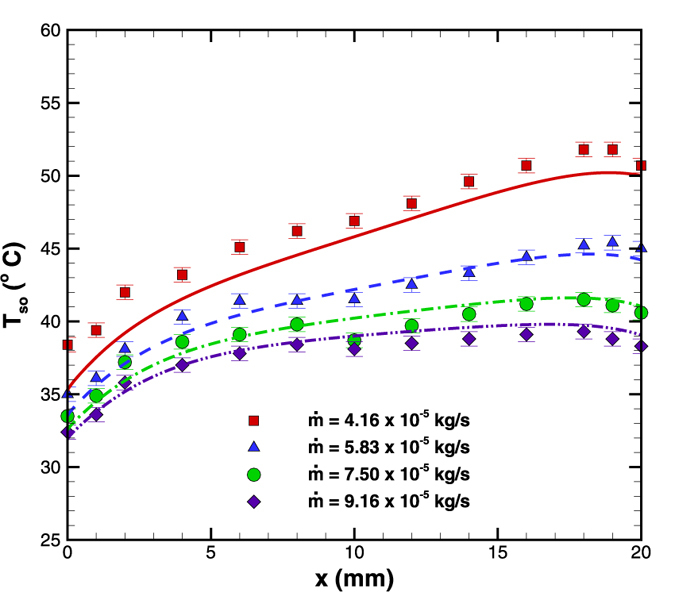
Surface temprature variation along flow direction in a diverging microchannel at q” = 4 W. (Note: symbols represent the experimental values whereas; lines show the corresponding numerical result).

Test section employed in experimental investigation is shown in Fig. 4 along with the schematic of experimental set up. The detailed geometrical configurations of test section are mentioned in Table 1. In order to make experimental measurements, the surface temperature was measured using a thermocouple positioned at different axial locations (marked in Fig. 4c); and the temperatures were recorded under steady state condition. The process was repeated over the entire length of the microchannel. Figure 5 shows the axial temperature variation for four different flow rates between 4.17 × 10^−5^ –9.17 × 10^−5^ kg/s (Re = 81–178). The reduction in surface temperature gradient is particularly evident at higher flow rates. Therefore, for the present microchannel configuration it is possible to reduce the surface temperature variation below 2 °C over a large part of the microchannel by maintaining 9.17 × 10^−5^ kg/s of water flow through a diverging microchannel.

**Figure 6:**
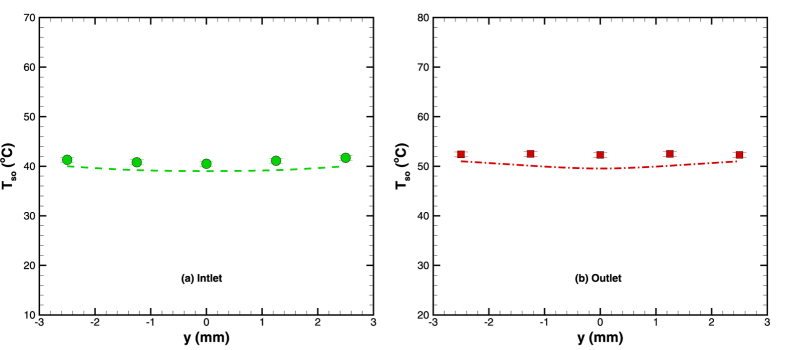
Comparison of lateral surface temperature variation in diverging microchannel at m = 4.17 × 10^−5^ kg/s and q_sup_ = 4 W (Note: symbols represent the experimental values whereas; lines show the corresponding numerical result).

In addition to the axial variation, lateral variation of the temperature was also measured at both inlet and outlet locations (Fig. 4c). From Fig. 6, it can also be seen that the surface temperature variations along the lateral direction were not significant; and the temperature can be more or less assumed to be constant along the transverse direction. The reason for such uniformity of temperature in the transverse direction is the fairly high thermal conductivity of the silicon substrate (148 W/m-K). Figures 5 and 6 also present comparison of experimental and numerical results; the two are clearly in good agreement.

**Figure 7:**
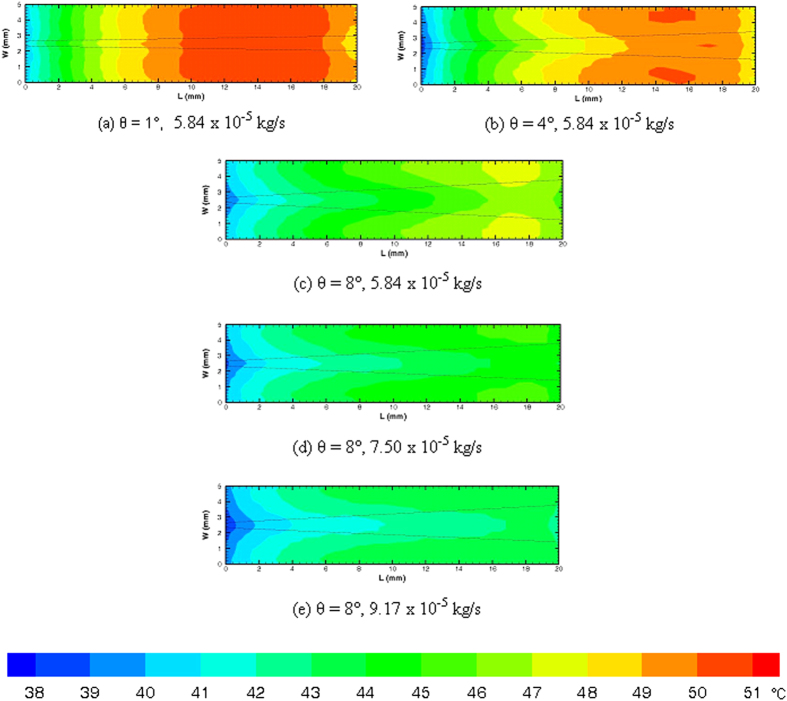
Experimentally measured temperature contours in diverging microchannels with varying divergence angle (**a–c**) at constant mass flow rate of 5.84 × 10^−5^ Cg/s and varying mass flow rate (c–e) in 8° diverging microchannel.

Apart from thermocouple measurements, thermal imaging is also performed using IR (Infrared camera) camera. Experiments are carried out on three different diverging microchannels with divergence angle of 1, 4 and 8° to compare the effect of divergence on surface temperature gradients. Inlet width (257 ± 17 μm) and depth (105 ± 5 μm) of all the microchannels are kept constant. All microchannels are supplied with constant heat input of 4 W. Figure 7 shows the variation of surface temperature with diveregnce angle (Fig. 7a–c) at a given mass flow rate of 5.84 × 10^−5^ kg/s. It can be seen that the temperature gradient reduces with increase in divergence angle from 1 to 8°. Further for 8° diverging microchannel increase in mass flow rate from 5.84 × 10^−5^ kg/s to 9.17 × 10^−5^ kg/s results in nearly isotherm condition (see Fig. 7c,e) with slight gradient near the inlet.

The above results point to the fact that the surface temperature gradients reduce significantly for diverging microchannels. It can therefore be concluded that by judiciously varying the thermophysical properties and input parameters, a nearly isothermal wall boundary condition can be obtained for a diverging microchannel. In the next section, a detailed parametric study analyzing the effect of different geometrical, flow and thermo-physical properties on the wall boundary condition is undertaken to establish the above finding over a larger parameter range.

### Effect of other parameters

A number of parameters affect the magnitude of axial back conduction and alteration in the heat flux distribution in the microchannel. These parameters are: geometrical (angle, length, depth, solid-to-fluid thickness ratio), thermo-physical (thermal conductivity ratio of solid-fluid), and input conditions (mass flow rate, heat flux). A detailed parametric study was carried out, as discussed under the subsequent subsections.

### Effect of angle

**Figure 8:**
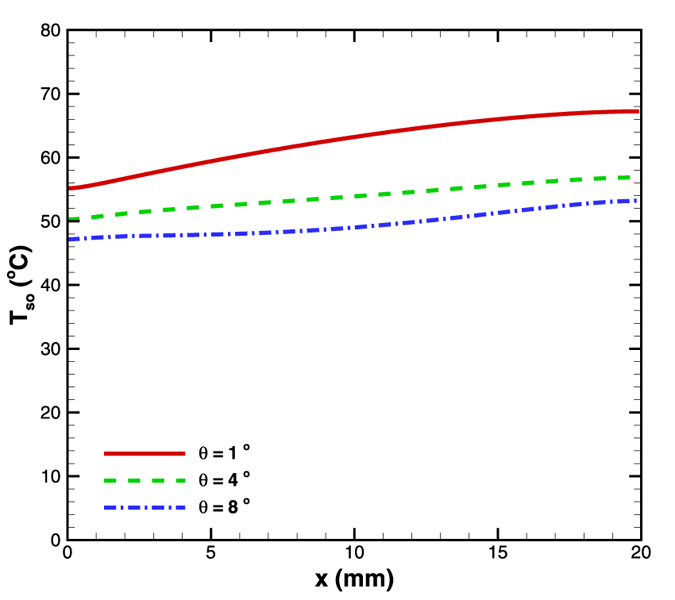
Effect of angle on surface temperature variation in diverging microchannel obtained numerically.

The three angles considered here are 1, 4 and 8^o^. The inlet width was fixed as 200 μm and simulations were performed for a mass flow rate of 5 × 10^−5^ kg/s with bottom of the substrate maintained at a constant heat flux of 4.8 W/cm^2^. The idea of redistribution in flux and its effect on obtaining isothermal conditions has been demonstrated successfully in previous section for 8^o^ diverging microchannel. As the angle was increased from 1^o^ onwards, there was relatively sharper increase in the area. So, the heat flux absorbed along the length of the microchannel as well as the Reynolds number gradients (thus Nusselt number) decrease steeply. Therefore, with increase in angle, the surface temperature gradients reduced to a great extent as evident from Fig. 8. For example, surface temperature gradients (ΔT_s_/L; where ΔT_s_ is the difference in surface temperature taken at inlet and outl *et al*ong the centerline as shown in Fig. 4c, and L is the length of microchannel) for 1, 4 and 8^o^ microchannel were observed to be 0.6, 0.35 and 0.3 °C/mm respectively.

The results therefore suggest that an increase in the angle leads to a reduction in the surface temperature gradient, and is therefore preferable. However, there is an upper limit to the angle (16^o^), beyond which flow reversal will come into the picture^22^; flow reversal would significantly alter the flow in the microchannel and the associated temperature and heat flux distributions.

### Effect of depth

**Figure 9:**
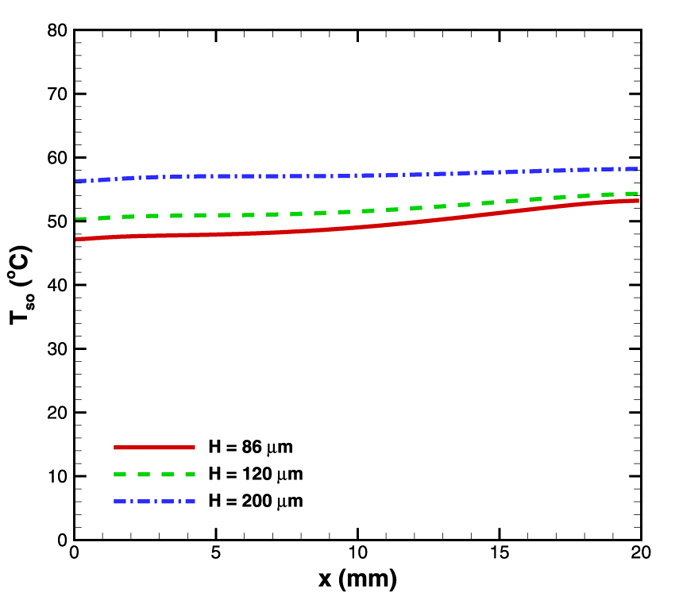
Effect of depth variation on surface temperature distribution in diverging microchannel obtained numerically.

Effect of microchannel depth was also analyzed by simulating for three different microchannel depths –86, 120 and 200 μm; while keeping the solid to fluid thickness ratio constant along with rest of the geometrical parameters. Again the flow rate was maintained at 5 × 10^−5^ kg/s and the wall heat flux as 4.8 W/cm^2^. Increasing the depth and maintaining a constant solid to fluid thickness ratio leads to a decreased Reynolds number (Re), which reduces the convection strength. As a result, the average surface temperature increases with increase in the microchannel depth, as can be observed from Fig. 9. Also, the surface temperature gradient is observed to decrease with depth. It can be observed that the gradients are 0.3, 0.2 and 0.1 °C/mm respectively for 86, 120 and 200 μm microchannels. This is because the reduction in Re with depth is more towards the narrower region. Therefore, the convection effect drops largely towards the narrower end, which leads to an increase in the surface temperature. On the contrary, towards the larger end the drop in Re is not much and hence an increase in the surface temperature at this end is small which brings in the constant wall temperature condition.

The microchannel depth therefore has an important effect on the temperature distribution; choosing a larger depth is advantageous from the point of view of maintaining a constant surface temperature.

### Effect of length

**Figure 10:**
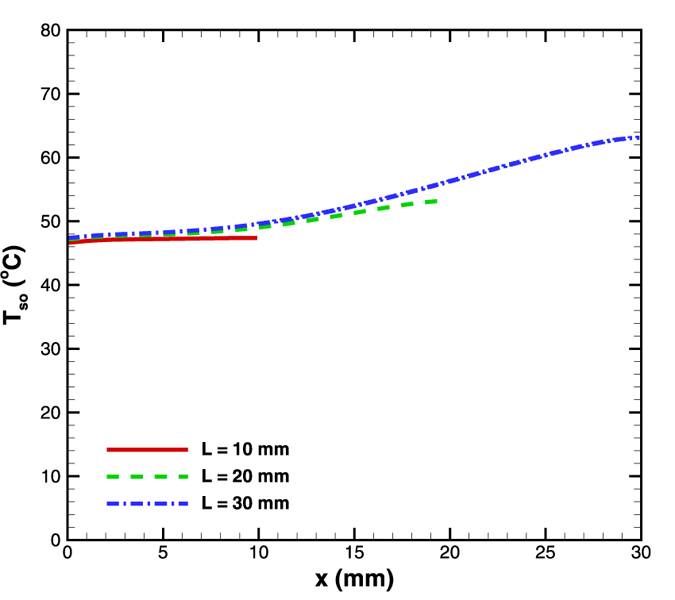
Effect of length variation on surface temperature distribution in diverging microchannel obtained numerically.

In the next set of simulations, the effect of microchannel length on the ensuing surface temperature gradient was analyzed. The microchannel lengths considered were 10, 20 and 30 mm with all other geometrical parameters remaining the same. The mass flow rate was maintained at 5 × 10^−5^ kg/s and the bottom wall was supplied with a constant heat flux of 4.8 W/cm^2^. Figure 10 shows a nearly isothermal surface condition (~1 °C) for 10 mm length microchannel. It was further observed that as the length reduced, the surface temperature gradient also reduced. For 10, 20 and 30 mm length microchannels, the temperature gradients were respectively 0.10, 0.30 and 0.53 ^o^C/mm. The reason behind this nature is the decrease in the convection effect with length. The decrease in Reynolds number for 30 mm length microchannel is substantially higher than that of 10 mm length microchannel. As a result the 30 mm length microchannel shows a larger surface temperature gradient owing to reduced strength of convection in the downstream direction.

This result leads to a significant conclusion that a shorter length leads to a more uniform temperature distribution. Having a uniform temperature over a small length is particularly useful for microdevices.

### Effect of solid to fluid thickness ratio

Conduction in solid becomes significant when the solid to fluid thickness ratio exceeds unity^18^,^19^,^20^,^21^. In order to examine the effect of solid to fluid thickness ratio on the surface temperature, the base case having t_s_/t_f_ = 2.32 was compared against t_s_/t_f_ = 1.5 and t_s_/t_f_ = 4. Microchannel depth was maintained constant as 86 μm while the solid thickness was varied appropriately to obtain the desired ratio. This was done while maintaining all other geometrical parameters same as the base case. Again, the heat flux was maintained at 4.8 W/cm^2^ and the mass flow rate was kept 5 × 10^−5^ kg/s for this set of simulations.

**Figure 11:**
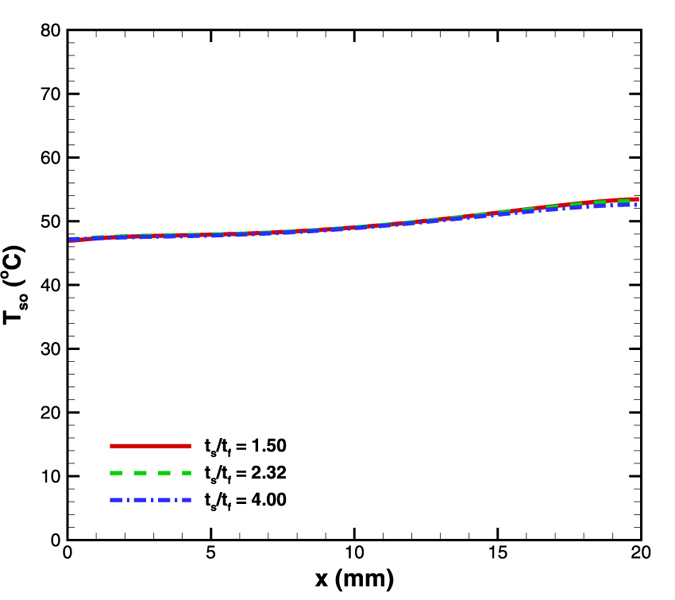
Effect of t_s_/t_f_ variation on surface temperature distribution in diverging microchannel obtained numerically.

It was observed that the surface temperature gradients reduce when t_s_/t_f_ increases owing to an increase in the strength of axial back conduction due to the increase in solid cross sectional area (Fig. 11). However for the range of values considered here, the difference in surface temperature gradient is insignificant. A drastic variation of t_s_/t_f_ is required to observe substantial change in the surface temperature distribution.

### Effect of solid to fluid thermal conductivity ratio

**Figure 12:**
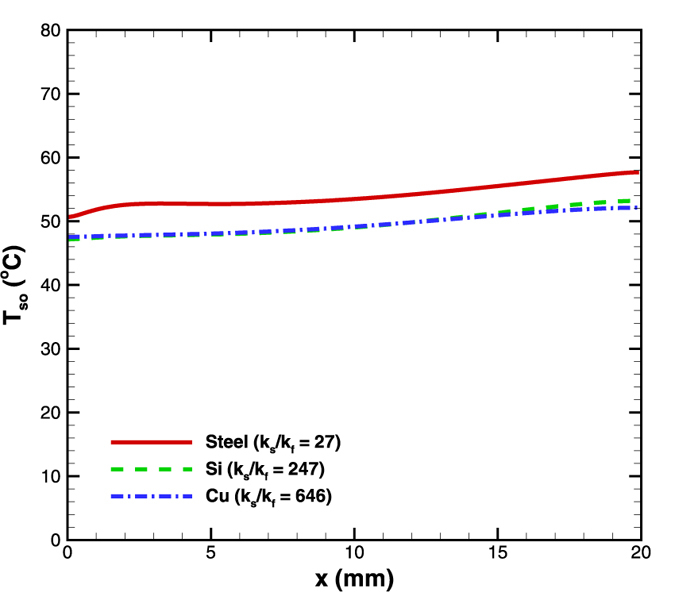
Effect of solid to fluid conductivity ratio on surface temperature variation in diverging microchannel obtained numerically.

Simulations were also performed to study the effect of solid conductivity (k_s_) to fluid conductivity (k_f_) ratio with working fluid fixed as water. For this purpose, the base case was taken as silicon (k_s_/k_f_ = 247) and the results compared against steel (k_s_/k_f_ = 27) and copper (k_s_/k_f_ = 646) as the microchannel substrate materials. The heat flux was again maintained as 4.8 W/cm^2^ and the mass flow rate as 5 × 10^−5^ kg/s. Since conductivity ratio is the only variable, it affects the intensity of axial back conduction. Increasing the conductivity ratio reduces the thermal resistance (L/k_s_A; where L is the microchannel length and A is the cross section area) to axial back conduction. Therefore, the average surface temperature reduces for silicon and copper as compared to steel (Fig. 12). The temperature gradients come out to be 0.35, 0.3 and 0.28 °C/mm for steel, silicon and copper, respectively.

The above results suggest that unless the conductivity ratio is varied drastically, a substantial effect on the surface temperature gradients will not be seen. That is, the results presented in this study using silicon wafer is equally applicable to many other solid-liquid combinations as well. Any material which does not react with the biological sample and has a good thermal conductivity can be employed to make microchannels, making this methodology all the more attractive.

### Effect of mass flow rate

**Figure 13:**
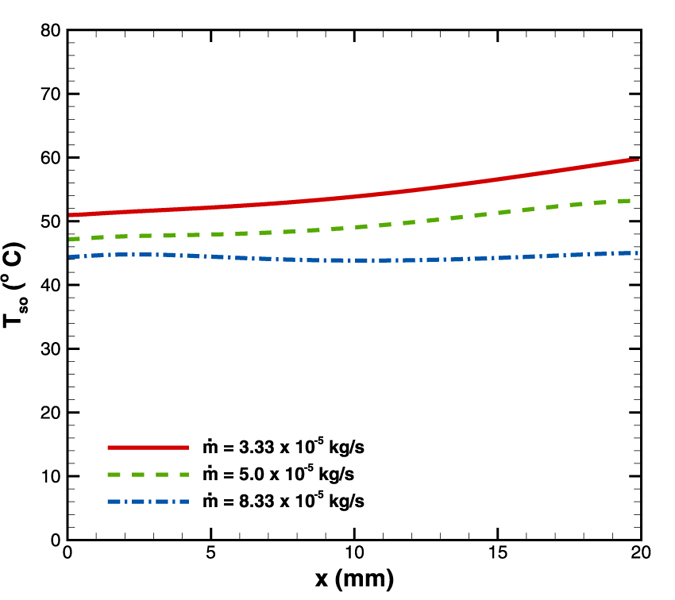
Effect of mass flow rate on surface temperature distribution in diverging microchannel obtained numerically.

Additional simulations were performed for 8^o^ diverging microchannels in order to observe the effect of mass flow rate on the temperature distribution at the bottom surface. The mass flow rates considered were 3.33 × 10^−5^, 5.0 × 10^−5^ and 8.33 × 10^−5^ kg/s (respectively 2, 3 and 5 ml/min) at supplied heat flux of 4.8 W/cm^2^ keeping all other geometrical parameters same. It is observed that the surface temperature gradient as well as the average surface temperature reduced substantially at higher flow rates (Fig. 13). The temperature gradients for 3.33 × 10^−5^, 5.0 × 10^−5^ and 8.33 × 10^−5^ kg/s are 0.45, 0.3 and 0.05 °C/mm respectively. At higher flow rates, the convection heat transfer becomes substantially higher than the wall conduction. Therefore the relative magnitude of wall conduction is reduced with an increase in the flow rate.

Hence, both lowering of the average surface temperature as well as the reduction of surface temperature gradient is brought about at higher flow rates. However, employing higher flow rate would lead to a higher pressure drop penalty and should therefore be avoided. The constancy of the wall temperature should rather be maintained by suitably altering some other parameter.

### Effect of heat flux

**Figure 14:**
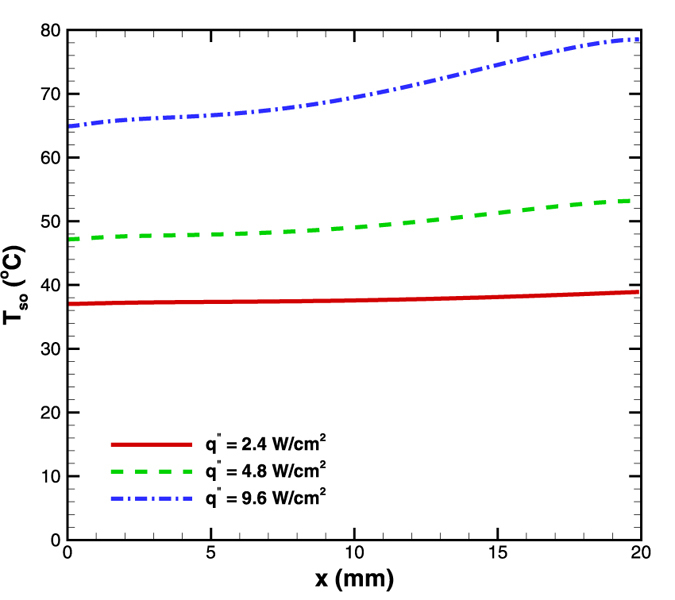
Effect of heat flux variation on surface temperature distribution in diverging microchannel obtained numerically.

Simulations were also performed in order to study the effect of heat flux supplied at the bottom wall. The mass flow rate of 5 × 10^−5^ kg/s was passed through a 8° diverging microchannel at three different heat fluxes of 2.4, 4.8, 9.6 W/cm^2^. The average temperatures went down with reduction in heat flux as expected (Fig. 14). Along with that, the temperature gradients were observed to be 0.1, 0.3 and 0.65 °C/mm for 2.4, 4.8 and 9.6 W/cm^2^, respectively. Again a near constant surface temperature was observed for lower heat flux. That is, a higher heat flux leads to a higher temperature in addition to a higher temperature gradient.

Combining the results of this section with the results from the section of Effect of length, it is noted that a lower heat flux over smaller segments of length is better than a higher heat flux over a larger length from the point of view of maintaining a nearly isothermal surface condition.

### Summary of the parametric study

**Figure 15:**
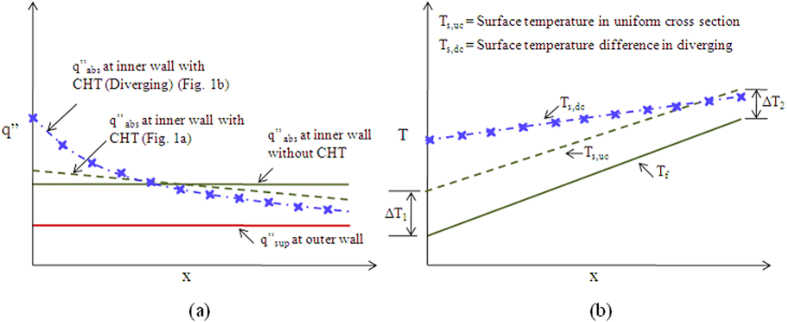
Schematic representation of (**a**) heat flux and (**b**) temperature variation along flow direction in diverging (blue line) and uniform cross sectional (green line) microchannel.

The experimental and numerical results showed that the combination of varying cross-section and wall conduction lead to a redistribution of heat flux which significantly alters the wall heat flux boundary condition. This also affects the temperature distribution in the microchannel. The idea of achieving constant wall temperature is reiterated through Fig. 15. The figure shows that heat flux distribution in uniform cross sectional microchannel becomes slightly non-linear in the presence of wall conduction (shown by dashed green line in Fig. 15a). This does not alter the temperature distribution in uniform cross sectional microchannel (shown by dashed green line in Fig. 15b). On the contrary, in diverging microchannel heat flux varies non-linearly because of the varying area (Fig. 15a). The non-linearity in heat flux further increases with wall conduction (shown by blue line in Fig. 15a), in such a way that the heat flux at the inlet is much higher than at the outlet. This leads to more heating of surface near the inlet compared to outlet surface (shown by blue line in Fig. 15b), leading to a nearly constant wall temperature condition.

A detailed parametric study was carried out to study the effect of different geometrical, thermo-physical and input parameters on the wall boundary condition which also provides an idea about the relative strength of convection and wall conduction in diverging microchannel. Table 2 presents a summary of the parametric study. It is observed that the temperature gradient (ΔT_s_/L) decreases asymptotically with divergence angle (Case I). This could be attributed to a reduction in Reynolds number and heat flux at inner walls in the downstream direction with increase in angle. In Case II, it is observed that the average surface temperature increases with depth of microchannel owing to a decrease in Re. However, ΔT_s_/L is observed to reduce with depth since the change in Re is more towards the narrower region as compared to the wider region. In Case III, the length of the microchannel was varied and it was found that a shorter length microchannel exhibits better surface temperature homogeneity (lower ΔT_s_/L). The reason for this is larger convection in shorter length microchannel (Re is higher) than in longer one with same angle of divergence. Also, there is a reduction in the resistance to the axial back conduction with decrease in length of microchannel. In the next two cases (Cases IV and V), the effect of wall conduction heat transfer on ΔT_s_/L was studied. Here, solid to fluid conductivity ratio and thickness ratio were varied and it was concluded that only a drastic variation in these parameters would have a significant bearing on the surface temperature variation. Furthermore, the effects of input parameters were studied. It was found that increasing the mass flow rate reduced the temperature gradient (ΔT_s_/L). Further simulations were performed by varying the substrate bottom wall heat flux and greater temperature homogeneity was observed when the heat fluxes were lower in magnitude.

In view of the detailed parametric study, the design parameters to achieve a nearly isothermal condition can be selected within the limit prescribed for the important parameters as: divergence angle (8–16°), depth (>86 μm) and length (<20 mm) of microchannel along with mass flow rate (>5 × 10^−5^ kg/s) and heat flux (<4.8 W/cm^2^). Hence, it can be concluded from the parametric study that under the set of optimal parameters, diverging microchannels would yield a near isothermal boundary condition (±0.5 °C).

### Model for Estimating the surface temperature

This section presents a model for estimating the surface temperature as a function of the various governing parameters. The role of various governing parameters in the direction of achieving isothermal wall condition has been identified in the previous section. Accordingly, a diverging microchannel with divergence angle: θ, depth: H and length: L is considered.

**Figure 16:**
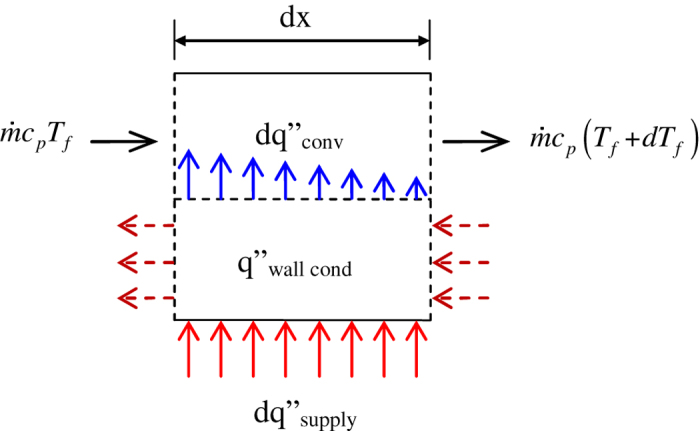
Control volume used in energy balance to derive the model for estimating the surface temperature.

Consider a control volume of diverging microchannel as shown in Fig 16. Using energy balance over the control volume we can write that the net energy entering (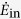) is equal to that of leaving (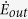) the control volume. Now, based on the above parametric study, it is assumed that most of the energy gets convected with the flow. We can write this as


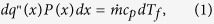


where, 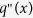 is heat flux supplied (W/m^2^), 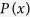 is the perimeter of diverging microchannel (m), 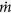 is the mass flow rate (kg/s), 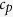 is the specific heat capacity (J/kg-K), and 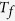 is local fluid temperature (K). Integration of Eq. 1 from inlet to outlet can be shown to give a linear variation of fluid temperature^9^.

Now consider a nearly isothermal wall boundary condition, Eq. 1 can be re-written as


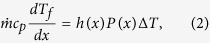


where 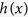 is the convective heat transfer coefficient (W/m^2^-K) and 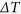
is the difference between surface and fluid temperatures, i.e. 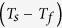. For nearly isothermal wall boundary condition we assume that 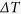
varies with the same rate as *T*_*f*_, i.e., 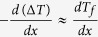. (This assumption is evaluated towards the end of the section.) Therefore, Eq. 2 becomes;


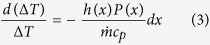


Eq. 3 can be integrated from inlet to outlet assuming that *h* is a constant calculated at the characteristic location for diverging microchannel^22^.

After integration and some algebra, Eq. 3 yields


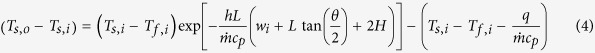


from which the surface temperature can be obtained as


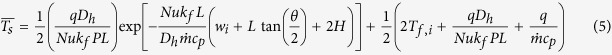


in terms of the relevant governing parameters.

Nusselt number (Nu) required in Eq. 5 can be obtained from a known correlation^23^ given by Eq. 6 below.


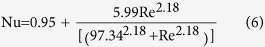


Reynolds number 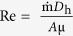 is calculated based on the hydraulic diameter 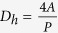 where, A, P are the cross sectional area and perimeter of diverging microchannel calculated at the characteristic location. The characteristic location lies at L/3 from the inlet^22^. The correlation for *Nu* used here is based on the experimental results for diverging microchannel and therefore accounts the effect of boundary layer on the prediction of surface temperature.

The surface temperature from Eq. 5 has been validated against experimental data in Table 3. The model clearly predicts the experimental data in the various cases tested to within 1 °C. The slightly higher value predicted by the model could be because we have neglected the effect of conduction heat flux in the model.

The above model, which has a strong theoretical basis, is therefore reasonably accurate. There are three main assumptions involved in the derivation of the model: (i) 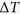
varies with the same rate as *T*_*f*_, (ii) *h* calculated at the characteristic location is representative over the entire microchannel, and (iii) 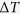 varies along flow direction (x-axis) only and its variation in lateral direction is negligible as shown earlier in Fig. 6. These assumptions are required to avoid unnecessary mathematical complexity without comprising the accuracy of the predictions. The effect of these assumptions is apparently small, at least for the parameter range considered here, as evident from the comparison in Table 3. We believe that these assumptions are not too severe even otherwise.

The model can be used to predict the temperature that the surface will attain for a given set of parameters. The model can further help in deciding the optimal design parameters of diverging microchannel in order to achieve a nearly isothermal boundary condition. The model was indeed utilized in this latter manner for PCR application presented in the next section.

### Applications and Significance of the design

In certain biological applications where maintaining a constant temperature (typically 37 °C) is of utmost importance in order to keep the cells alive, there is a need to design portable systems which can maintain the desired temperature^1^,^2^. Our results show that this is possible and the desired temperature can be maintained within ±0.5 °C. In order to achieve the same objective, researchers^6^,^7^,^14^ have proposed ingenious albeit intricate ways. The appeal of the proposed method lies in its simplicity. *By allowing a liquid to flow through a diverging microchannel supplied with constant heat flux at its bottom wall, a constant temperature can be achieved at the microchannel walls*. The proposed design therefore adds portability to the existing processes by eliminating the need of an incubator. This also suggests that for a given geometry and properties of diverging microchannel, *any constant wall temperature* can be obtained by changing the input parameters appropriately (i.e. with the help of Eq. 5).

**Figure 17:**
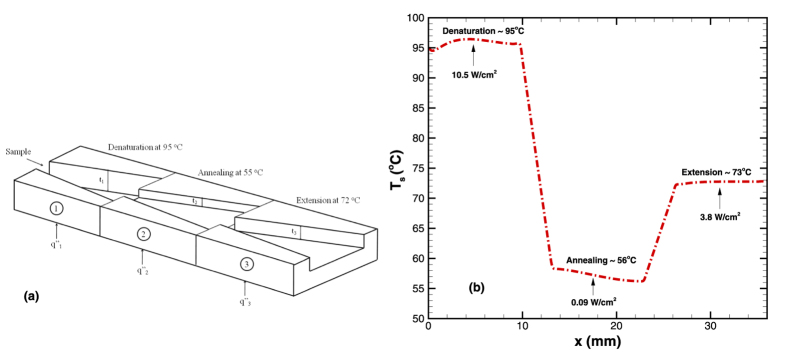
(**a**) Schematic representation of an on chip PCR process device consisting of three diverging microchannels with different geometrical configurations, (**b**) Numerically obtained temperature profile in three diverging microchannels arranged in series for PCR application.

We now demonstrate our concept on a series of microchannels which is relevant for a more complex example of PCR. The objective here is to maintain the different stations at the required temperatures of 95, 55, 72 °C (as explained in the Introduction section) so that PCR can be effectively carried out on a single chip. Three thermally insulated differently optimized diverging microchannels connected in series are employed for this purpose (Fig. 17a). The microchannels have different depths and different input heat fluxes. All the geometrical and input parameters are selected based on our model (Eq. 5). The results are obtained numerically and presented in Fig. 17b. The results are extremely encouraging in that three constant temperatures (~95, 57 and 73 °C) can be achieved in the three diverging microchannels with heat flux of 10.5, 0.09 and 3.8 W/cm^2^, for mass flow rate of 5 × 10^−5^ kg/s. The proposed design is therefore an important step forward in successful implementation of various lab-on-chip technologies.

### Methodology Employed

A brief description of the experimental and numerical methodologies employed in the present work is presented in this section.

### Experimental Investigation

Figure 4a presents a schematic of the experimental setup employed in the measurements^22^,^24^,^25^. The experimental setup consists of separate water and electrical circuits. The pump (Master Flex) used for pushing DI water through the microchannel provided volumetric flow rate ranging from 0.1 to 6 ml/min (1.67 × 10^−6^–1.0 × 10^−4^ kg/s). A pressure gauge (Keller Leo Record) equipped with data acquisition system recorded the pressure drop across the microchannel. A similar data logging arrangement (Graphtec Midi Logger) was employed for recording temperatures. The temperatures were measured by K-type thermocouples (Anbe SMT Co.) with bead size of 25 μm placed at the inlet and outlet of the microchannel as well as on the bottom surface of the microheater.

The microchannels employed for the purpose of experimental investigation (Fig. 4b,c) were fabricated on silicon wafer using MEMS techniques, involving photo lithography and wet etching; using in-house fabrication facilities. The detailed fabrication process is discussed elsewhere^24^. A thin meander type platinum micro heater of thickness 120 nm is deposited on the back side of the microchannel using metal sputter process (Fig. 4c). Resistance of the micro heater at room temperature is measured, as in^24^, to be ~1 kΩ. The values of different geometrical parameters of the microchannel along with the associated uncertainties are provided in Table 1. Heat supplied to the heater is utilized to heat the water flowing inside the diverging microchannel. However, the fraction of heat is lost to the atmosphere by convection and radiation. A standard method is utilised to quantify the amount of heat loss in the atmosphere. The percent heat loss is found to be maximum 12% at the lowest flow rate of 3.33 × 10^−5^ kg/s employed in the study. The detailed heat loss analysis is carried in Duryodhan *et al.*^23^.

### Numerical Investigation

**Figure 18:**
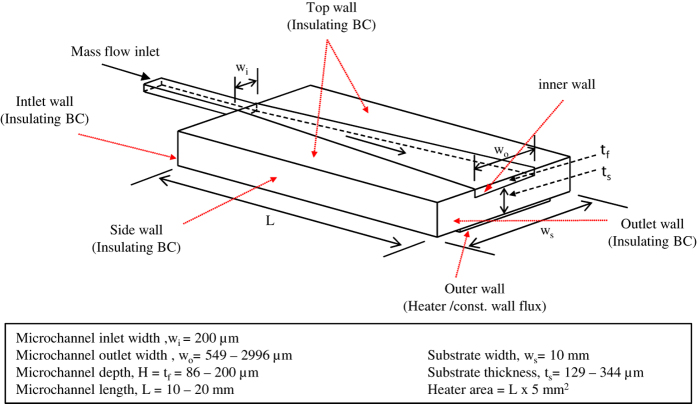
3D model employed in numerical simulation of diverging (8°) microchannel with the range of various parameters (not to scale).

In order to obtain additional local information of the relevant parameters, three-dimensional numerical simulations were performed. For this purpose a microchannel having the same dimensions as that of the experimental test section was modeled numerically. A straight section having length of 5 mm was placed before the inlet to ensure that the flow is hydrodynamically fully developed before entering the microchannel. The silicon substrate thickness and length of the microchannel were taken as 286 μm and 20 mm respectively. The 1 μm thick microheater was also modeled. Figure 18 shows the microheater-substrate-microchannel assembly; the 5 mm straight reservoir towards the bigger width size is however not shown in the figure. Specified mass flow rate was allowed to flow from the inlet of microchannel with outlet maintained at constant pressure condition. A constant heat flux was supplied at the bottom wall of the heater whereas all other walls are insulated. All the boundary conditions used in the numerical simulations are shown in Fig. 18.

The numerical simulations were performed using commercial package Fluent. The variation of fluid viscosity (μ) and thermal conductivity (k) with temperature (T) have been considered in the simulations. These are modeled as per the equations given below^9^:


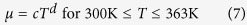


where c = 1.1 × 10^11^ and d = −5.7. Note that the viscosity of water decreases by about 69% with increase in temperature, over the temperature range considered. The thermal conductivity however increases linearly with increase in temperature, and was modeled as


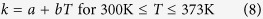


where a = 0.0033, b = 0.0019. The increase in thermal conductivity is 11% over the temperature range considered.

Numerical simulations were carried out by solving steady state governing equations (continuity, momentum and energy) for the three-dimensional geometrical model shown in Fig. 18. Semi Implicit Method for Pressure Linked Equations (SIMPLE) algorithm was employed to solve the steady state flow equations. Second order discretization scheme was employed for pressure equation; the momentum and energy equations were discretized using quadratic upstream interpolation for convective kinematics (QUICK). A grid independence test was systematically carried out and accordingly 450,000 cells were selected for the simulations. The Nusselt numbers from the simulations were validated against the experimentally obtained value for a number of cases; the two agreed within 7%. The Nusselt number is observed to vary from 3 to 5.6 for the range of Re employed in the present study^23^. Further validation of local temperature values is discussed through Figs 5 and 6 presented earlier. The simulations are therefore deemed to be validated.

## Conclusions

In this study, we show that microchannel of varying cross section brings about redistribution in heat flux; also wall conduction (or conjugate effect) comes into picture at microscales. These effects can be exploited to our advantage to obtain a constant wall temperature surface condition. Heating in microchannels was studied numerically and experimentally in order to observe the effect of redistribution in heat flux and the ensuing surface temperature gradient. A comparative study performed between straight and 8^o^ diverging microchannels showed a significant reduction in the surface temperature gradient for the latter case, lending credence to our proposition.

The proposed idea was also verified experimentally in an 8^o^ diverging microchannel over a wide range of flow rates. The lateral distribution of temperature showed an insignificant variation while the axial temperature gradient reduces substantially; thus implying that the surface temperature is almost constant. However, a diverging passage does not automatically imply a constant wall temperature condition. A detailed parametric study of the effect of various geometrical (angle, depth, length and solid-to-fluid thickness ratio), thermo-physical (thermal conductivity of solid) and input parameters (mass flow rate and heat flux) on the microchannel surface was undertaken to explore this finding over a wider parameter range. The outcome of this parametric study suggested the selection of important parameters as: divergence angle (8–16°), depth >86 μm, length <20 mm; along with mass flow rate >5 × 10^−5^ kg/s and heat flux <4.8 W/cm^2^. For this set of optimal parameters, diverging microchannel would yield a nearly isothermal boundary condition with temperature difference between inlet and outlet of less than 1 °C.

A mathematical model is also proposed to obtain the optimal geometrical parameters for a given set of input parameters (mass flow rate and heat flux). Eq. 5 derived in the paper can be employed for the purpose of designing the microchannel.

This constant temperature boundary condition in a diverging microchannel can be effectively utilized in certain biological applications where the samples are highly sensitive to temperature fluctuations. Finally, a successful demonstration of the proposed design is shown numerically which can facilitate development of an on-chip PCR. The simple and novel approach presented here can be employed in several other applications as well.

## Additional Information

**How to cite this article**: Duryodhan, V. S. *et al.* A simple and novel way of maintaining constant wall temperature in microdevices. *Sci. Rep.* **6**, 18230; doi: 10.1038/srep18230 (2016).

## Figures and Tables

**Table 1:** Geometrical specification of the microchannel employed in the present study.

Feature	Size	Uncertainty	Units
Smaller Width (W_i_)	270	+ 2	μm
Larger Width (W_o_)	3052	+ 2	μm
Length (L)	20	−0.1	mm
Height (H)	86	± 1	μm
Divergence Angle (θ)	8	0.5	°
Hydraulic Diameter (D_h_)	157	1.33	μm

**Table 2:** Summary of Parametric study showing dependency of ΔT/L on each parameter.

	Wall Temperaturegradient	Non dimensional GeometricalParameters					InputParameters		Remarks	
**(Case) Parameters**	**ΔT**_**s**_**/L (°C/mm)**	**θ**	**w/H**	**L/H**	**ts/H**	**ks/kf**	**m (kg/s)**	**q″ (kW/m**^**2**^)		
(I) Effect of Angle	0.875	0	2	233	2.33	247	5 × 10^−5^	48	Strong function	**Convection**
	0.600	1	4	233	2.33	247	5 × 10^−5^	48		
	0.350	4	8	233	2.33	247	5 × 10^−5^	48		
	0.300	8	13	233	2.33	247	5 × 10^−5^	48		
(II) Effect of depth	0.300	8	13	233	2.33	247	5 × 10^−5^	48	Strong function	
	0.200	8	9	167	2.33	247	5 × 10^−5^	48		
	0.100	8	6	100	2.33	247	5 × 10^−5^	48		
(III) Effect of length	0.100	8	8	116	2.33	247	5 × 10^−5^	48	Strong function	
	0.300	8	13	233	2.33	247	5 × 10^−5^	48		
	0.533	8	19	349	2.33	247	5 × 10^−5^	48		
(IV) Effect of thickness	0.300	8	13	233	1.50	247	5 × 10^−5^	48	weak function	**Conjugate/** wall conduction
	0.300	8	13	233	2.33	247	5 × 10^−5^	48		
	0.300	8	13	233	4.00	247	5 × 10^−5^	48		
(V) Effect of Thermal conductivity	0.275	8	13	233	2.33	646	5 × 10^−5^	48	Moderate function	
	0.300	8	13	233	2.33	247	5 × 10^−5^	48		
	0.350	8	13	233	2.33	27	5 × 10^−5^	48		
(VI) Effect of Mass flow rate	0.450	8	13	233	2.33	247	3.33 × 10^−5^	48	Strong function	**Convection**
	0.300	8	13	233	2.33	247	5.0 × 10^−5^	48		
	0.050	8	13	233	2.33	247	8.33 × 10^−5^	48		
(VII) Effect of Heat flux	0.075	8	13	233	2.33	247	5 × 10^−5^	24	Strong function	
	0.300	8	13	233	2.33	247	5 × 10^-5^	48		
	0.650	8	13	233	2.33	247	5 × 10^−5^	96		

**Table 3:** Validation of proposed model with experimental data (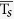 denotes average surface temperature).

Mass flow rate (kg/s)	Power Supplied (W)	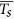 , °C (Experimental)	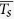 , °C (Proposed model Eq. 5)	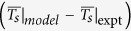 , °C
3.33 × 10^−5^	3.5	49	50	1
5.00 × 10^−5^	3.5	44	45	1
6.67 × 10^−5^	3.5	41	42	1
8.33 × 10^−5^	3.5	39	41	2
10.0 × 10^−5^	4.0	40	41	1
